# Recognition of Rare Low-Moral Actions Using Depth Data

**DOI:** 10.3390/s20102758

**Published:** 2020-05-12

**Authors:** Kanghui Du, Thomas Kaczmarek, Dražen Brščić, Takayuki Kanda

**Affiliations:** Graduate School of Informatics, Kyoto University, Kyoto 606-8501, Japan; thomas@robot.soc.i.kyoto-u.ac.jp (T.K.); drazen@i.kyoto-u.ac.jp (D.B.); kanda@i.kyoto-u.ac.jp (T.K.)

**Keywords:** human action recognition, depth maps, skeleton, low-moral actions, 3D CNN

## Abstract

Detecting and recognizing low-moral actions in public spaces is important. But low-moral actions are rare, so in order to learn to recognize a new low-moral action in general we need to rely on a limited number of samples. In order to study the recognition of actions from a comparatively small dataset, in this work we introduced a new dataset of human actions consisting in large part of low-moral behaviors. In addition, we used this dataset to test the performance of a number of classifiers, which used either depth data or extracted skeletons. The results show that both depth data and skeleton based classifiers were able to achieve similar classification accuracy on this dataset (Top-1: around 55%, Top-5: around 90%). Also, using transfer learning in both cases improved the performance.

## 1. Introduction

In public spaces, people exhibit a variety of behaviors. Although the majority of actions that we can observe are socially appropriate, there are also actions that are regarded as socially inappropriate. For example, actions that break a written law or rule of conduct, such as littering, stealing, or purposefully injuring other people, or actions that break “unwritten rules“ defined by the social norms of a community, like talking too loud or forcing one’s way through other people, are seen as inappropriate. We refer to all such actions as *low-moral actions*. What exactly constitutes a low-moral action is highly dependent on the community and environment where the action happens. However they generally cause nuisance for other people in the public space, so being able to detect a low-moral action when it occurs would be beneficial. For example, it could help security personnel intervene early and warn or stop the wrongdoer (or, this could also be done by a robot, as in [1]).

Recognition of human actions is an area of study which has seen great progress recently. In particular, advances in neural network architectures and the increasing availability of data has enabled the development of methods that achieve highly accurate classification of many different actions, see e.g., [2], etc. These recognition methods presuppose the existence of a large number of labeled samples of the actions that we wish to detect. Thanks to the efforts of several research groups, a number of such large datasets of actions exist today, e.g., [3,4,5,6]. Nevertheless, the types of actions that these datasets provide are limited to the ones chosen by the researchers that collected them, and in general only a few of them are low-moral actions (as an example, [7] contains 120 different actions, but only about 10 can be considered low-moral ones, e.g., kicking or pushing).

In order to detect other types of actions it is necessary to collect new samples. But low-moral actions are, by their nature, rare in public spaces. Thus, if we wanted to collect a large number of samples directly from public spaces, considerable time and effort would be needed. The alternative is to invite participants in the lab and ask them to act out the behaviors, like it was done e.g., in [6], but that too requires an extensive effort. Instead of that, what we wished to understand in this work is what can be done if we have only a relatively small set of action samples. More precisely, we were interested in practical questions related to this case, such as: which data representation is better, will state-of-the-art classifiers perform well, and are techniques like transfer learning useful.

Human action recognition is nowadays most commonly done using RGB camera streams. However, due to the concern with personal privacy in public spaces, it is often difficult to obtain permission for collecting data with RGB cameras. As an alternative, 3D range sensors (depth cameras) are sometimes used for action recognition. The measurement they give is a stream of images, in which, instead of RGB color, each pixel gives the distance to an object in that particular direction—The resulting image is often called a “depth map“. Compared to an RGB image, identifying a person in a depth map is generally much harder. An alternative approach is to extract a skeleton representation of a person from depth data (such as in [8]), and to use that for recognizing the action.

In this work we were concerned with utilizing depth maps or extracted skeleton data for detecting actions for which only limited data can be collected, such as low-moral actions. The main contributions of this work are as follows:

- We have collected a novel dataset of human actions, named *Low-Moral Actions (LMA) Dataset*. It includes several low-moral actions, collected both as depth maps and as skeleton data.
- We evaluated the action recognition performance on this dataset using a number of representative approaches used for large datasets: a new 3D convolutional neural network based classifier for depth data as well as state-of-the-art classifiers for skeleton data. Moreover, we tested the use of transfer learning for improving the recognition performance on comparatively small datasets.

## 2. Related Work

### 2.1. Human Action Recognition Using Depth Data

Many researchers have been using depth sensor data for human action recognition. One survey which covers action recognition methods using depth maps and skeleton data (as well as RGB data) is presented in [9]. A recent paper by Lei Wang et al. [10] reviews and compares the performance of several state-of-the-art algorithms for both depth map and skeleton data representation.

*Depth map based*: One approach to recognizing the human action is to use the raw depth map data directly. Several previous works used hand-crafted features, such as surface normals [11], space-time volume [12], or silhouette information [13]. Another common feature is the “depth motion map“ (DMM), which combines a sequence of depth maps into a single 2D image [14]. A survey of feature-based methods can be found in [15]. More recently there have been several works that use convolutional neural networks (CNN). CNNs have shown great performance on action recognition tasks using RGB data as well as RGB-D (i.e. RGB and depth together) data [9]. Pichao Wang et al. published a number of works where they used as input to a CNN some variant of DMM [16,17] or other types of 2D image features [18]. A different approach, which we used in this work, is to treat the sequence of depth maps directly as 3D input (dimensions are image depth and width, and time) and use a 3D CNN as classifier (as first proposed in [19] for RGB videos). For example, Wu et al. [20] used 3D CNNs for recognizing gestures in RGB-D data. Compared to standard 2D CNNs, 3D CNNs have more parameters and thus require large datasets to train, which is why there is still little research on their use. To address this issue, Zhang et al. [21] recently proposed several light-weight 3D CNN structures and shown that they achieve good performance on a number of datasets, albeit using RGB-D data. In this work we followed a similar approach to [21], and developed a 3D CNN classifier for action recognition that uses only depth data.

*Skeleton based*: A different approach to recognizing human actions from depth data is to first extract the 3D human body joint positions from the depth images. The resulting skeleton gives a compressed representation of the human pose which simplifies the recognition. Since several depth sensors on the market provide easy ways to extract skeleton data, recently there has been a wealth of research on detecting human actions based on skeleton information. Similar to depth data, earlier works relied on hand-crafted features, e.g., [22]. However, the current predominant approach is to use the whole skeleton directly as input to some variant of a neural network. A number of works employed recurrent neural networks (RNN) or long–short-term memory (LSTM) structures to model the spatial-temporal evolution of the skeleton joints [6,23,24,25]. A different approach is to treat the skeleton data as a pseudo-image, which turns the action recognition into a image based classification problem, for which convolutional neural networks (CNNs) can be used [26,27,28,29].

By simply representing the skeleton data sequences as images, the intrinsic relationships between adjacent joints and bones are not taken into account. Therefore more recent works model the skeleton data as a graph, with joints as vertexes and bones as edges, and then apply graph convolutional networks (GCN) [30] to perform the action recognition. In fact, at this moment essentially all methods with top recognition performances on large datasets such as NTU RGB+D are based on graph convolutional networks (According to the statistics on the Papers With Code site [31]). Yan et al. [32] were the first one to propose one such classifier, named Spatial Temporal Graph Convolutional Network (ST-GCN). A number of improvements on ST-GCN have been proposed, such as combining both actional and structural links into a generalized skeleton graph (AC-GCN) [33], using directed graphs (DGNN) [2], or handling action recognition jointly with motion prediction (Sym-GCN) [34]. The recent top-performing method is MS-G3D [35], which introduced a way to remove redundant dependencies between different node neighborhoods, as well as a unified spatial-temporal graph convolutional operator called G3D. In this work we used two representative approaches for evaluation on our dataset: ST-GCN, as the standard baseline GCN structure, and DGNN, which was the top performing method at the time we started the research.

Several researchers have also used a combination of both depth maps and extracted skeleton data [36,37,38]. However, in this work we focused on the use of depth maps and skeleton data separately and did not test their combination.

While the majority of the works listed here was concerned with the improvement of action recognition accuracy on an existing dataset, we were interested in finding out the best achievable performance on our newly introduced dataset. Therefore in this work we chose several representative top-performing approaches and tested them on the dataset.

### 2.2. Recognition of Rare Actions

A topic closely related to our work is the detection of behavior which is atypical or abnormal, where the aim is to detect human actions that are different from the ones that can typically be observed (suspicious, irregular, uncommon). References [39,40] provide thorough reviews of the existing research. However, in general the main concern in those works is only to detect if an action is atypical, but not to recognize what that action actually is, which is what we wanted to achieve.

One exception to that is the detection of fall situations, which has received considerable attention [41,42]. Several datasets of falls, taken with different sensing modalities, are available [43,44]. Most of the research works concentrated on the improvement of detection accuracy on an existing dataset. Some researchers have also tried to addressed the issue of having a small training dataset by using approaches like transfer learning [45,46]. Although that is related to what we did in this work, the classification problem they were solving was binary (person fell or not), and they did not do a comparison of multiple approaches.

Another line of research on detecting actions which are rare are the works on one-shot learning [47]. In one-shot learning the aim is to learn to recognize a class using only one or a few training samples. (When more than one sample is used this is often called few-shot learning.) There is still relatively little work on one-shot learning for action recognition, especially for depth data. For example, Refs. [7,48] describe the results of one-shot learning on RGB-D data. An interesting approach to action recognition from depth data is presented in Guo et al. [49], where they generate a graph of the spatial relationships in the scene (including objects) and a corresponding graph matching metric. Both parts are jointly trained, which enables them to directly optimize for few-shot learning of novel actions. We did not consider one-shot learning techniques in this work, but that may be an interesting direction for future studies.

Transfer learning and data augmentation are an often used techniques for learning on small datasets. The main idea of transfer learning is to pretrain the classifier on a related large dataset and use that as a starting point for training on the smaller target dataset. Very few works used transfer learning to improve the action recognition on depth or skeleton data. Pham et al. [29] used pretraining on large action datasets to improve the performance of skeleton based action recognition on their smaller dataset of passenger behavior in a metro station (the dataset included also low-moral actions like jumping over or sneaking under ticket barriers). In this work we used transfer learning in a similar way, and tested it for both depth and skeleton data based action recognition.

Only a small number of works explicitly studied the detection of low-moral actions. That is particularly true for depth data since not many datasets for that purpose are publicly available. One example of research on low-moral action recognition is the just mentioned metro station study in [29]. Another is [50], where the authors recognized the gestures of children bullying a robot using extracted skeleton data.

## 3. Low-Moral Actions Dataset

### 3.1. Experimental Setup

**Figure 1:**
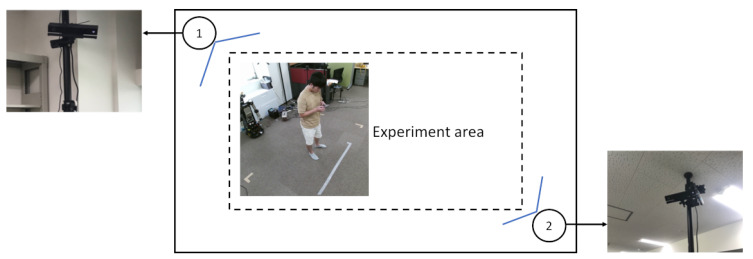
An illustration of the experimental setup. The circles represent the positions of the Kinect sensors. The size of the experiment area is about 2.8 × 1.5 m.

We set up a data collection environment using 2 Microsoft Kinect v2 sensors. The size of the experiment area was approximately 2.8 × 1.5 m, see Figure 1. Two Kinect sensors were placed in the opposite corners of the rectangular experimental area. They were mounted on poles at the height of about 2.2 m, facing down at an angle of approximately 45${}^{\circ }$. With this configuration both sensors could observe the people who were inside the area.

The Kinect sensors output both RGB and depth data, which can be saved using the Kinect for Windows Software Development Kit 2.0. In addition, the Software Development Kit allows the extraction of the skeletal representation of the people in the area observed by the sensor.

### 3.2. Collected Actions

**Figure 2:**
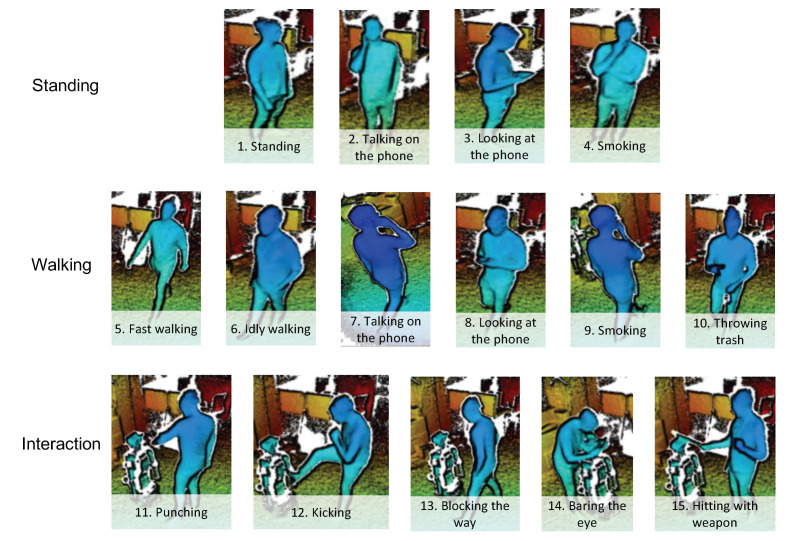
Collected actions in the Low-Moral Actions (LMA) dataset.

The 15 actions that we collected are shown in Figure 2. The actions in the Low-Moral Actions (LMA) dataset are divided into three groups: actions done while standing, actions done while walking, and interactive actions. The first two groups contain both typical daily actions, like simple walking and standing, as well as several low-moral actions, such as smoking, walking while playing with the phone, etc. The last group includes behaviors that happen during interactions, in particular actions that can be considered low-moral (the contained actions are the ones that we observed during our real-world study of abusive behavior toward robots in [51]).

An important feature of this set of actions is that it has a relatively small overlap with existing datasets. For example, the percentage of action types in the LMA dataset that is also contained in the 60 actions in the NTU RGB+D dataset [6] is 33%, and in the 120 actions of the NTU RGB+D 120 dataset [7] is 40%. To compare this with two other available datasets that provide both depth and skeleton data, for the MSRAction3D dataset [3] the percentages are respectively 50% and 70%, and for the UWA3D Multiview Activity II dataset [5] 70% and 77%.

We invited 20 people to the lab to act out the behaviors. The ages of the subjects were between 22 and 54 (12M, 8F). We only gave the subjects a short name and one-sentence explanation of each action. In order to obtain more variations we did not give specific details how they have to do the actions, but let them perform the actions in the way they liked. During that time we recorded them using Kinect sensors. For actions that included walking, the subjects would walk from one edge of the experimental area to the other, then do a quick turn and walk in the other direction. For easier acting of the interactive actions, we placed a robot in the area (see third row in Figure 2). However, we asked the subjects to do the punches and kicks such that they do not get hurt or break the robot (they stopped their hits mid-air or just lightly touch to robot).

Each action sequence was recorded for about 13 s, and every participant acted out the same action 2 times. After discarding some of the data due to problems with saving we collected in total 1166 sequences of depth maps. In a number of runs the subjects exited the experimental area and were not well visible in one of the sensors, so the skeleton data could not be continuously collected. These sequences were discarded and as a result we had in total 1064 full 13-s sequences of skeleton data.

The data collection required effort, and with about 80 samples per action class the LMA dataset cannot be called tiny. Nonetheless, its size cannot be compared to a large dataset like NTU RGB+D [6], which has 3200 samples for each of the 60 actions.

The LMA dataset is made freely available. For the URL see Supplementary Materials.

## 4. Action Recognition Methods

We tested several approaches for recognition. In particular, we compared approaches that differ along the following dimensions:

- Representation of input data: either full depth map or extracted skeleton of person;
- Basic approach to training: training from scratch or using transfer learning;
- Dataset for the classifier pretraining (for transfer learning)

### 4.1. Depth Map Based Recognition

#### 4.1.1. Representation: Depth Maps

**Figure 3:**
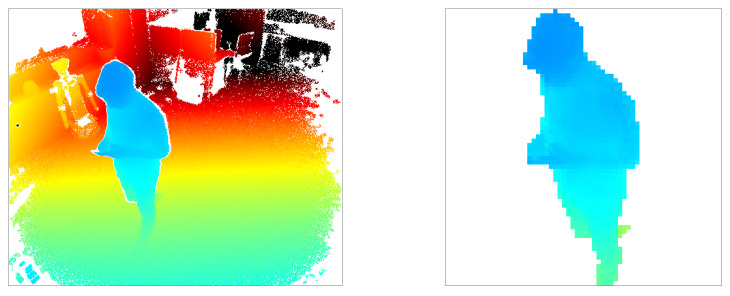
Example data maps before and after preprocessing: (**left**) original 512 × 424 depth map from Kinect; (**right**) transformed 64 × 64 depth map containing only the parts belonging to person.

The first representation of the data that we used is the depth map, i.e. the raw measurement output of the depth sensor. In depth maps, each pixel in the image represents the distance to the camera. (Each pixel contains one value, so a depth map is similar to a grayscale image. Nevertheless, on Figure 2 ans Figure 3 and later on Figure 5 we mapped the distance to colors for better visualization).

Many of the pixels in the depth map represent the background (floor, wall, and other static objects) so they are not relevant for the action recognition task. Therefore we created the depth image of the background containing only the static objects, and then extracted the pixels which were not contained in the background. The resulting foreground represents the person in the image. The foreground pixels were adjusted (by zooming in or out if necessary) to closely fit into an image with a fixed size of 64 × 64 pixels. Figure 3 shows an example of an original depth map and the transformed 64 × 64 image after background extraction and resizing to fit the observed person.

For recognizing the action we used 5-s sequences of the transformed depth maps. Kinect sensors output 30 depth maps per second, but using all 150 frames makes the classifier training very slow. Instead, we selected every 5th frame, which gave us 30 frames in total. Therefore, for depth data the input to the classifier was 30 frames of 64 × 64 depth maps.

#### 4.1.2. Training Approaches

Training from Scratch

Training from scratch is a straightforward approach to train a classifier—i.e., starting with randomly initialized parameters of the classifier and learning their values based on the training data.

**Figure 4:**
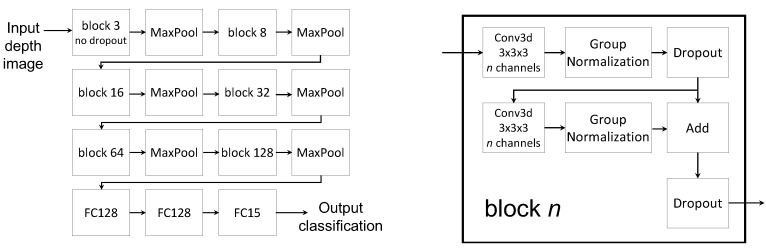
The neural network structure used for the depth map based classification (DM3DCNN).

We treated the input data as three-dimensional – image width and height, plus time (frame). Thus we needed a classifier to process 3D data. We used a variant of a convolutional neural network (CNN) [52], with the structure shown in Figure 4. The structure is similar to commonly used CNN structures, except that it uses 3D instead of 2D convolutions, plus a number of improvements to obtain better performance and make it light-weight. We refer to this classifier as DM3DCNN (“Depth Map 3D CNN”).

The right side of the figure shows the main building block of the convolutional part of the neural network. The block consists of a 3D convolution operation followed by group normalization [53] (an improved alternative of batch normalization) and dropout. This is followed by another 3D convolution and group normalization in parallel with a skip connection, and finally ending with dropout. The full network structure, shown on the left side of Figure 4, consists of a sequence of 6 of the previously described 3D convolution blocks followed by 2 × 2 × 2 max pooling operations. The network ends with 3 fully connected layers. We used scaled exponential linear units (SELUs) [54] as activation functions for both the convolution and the fully connected layers, except for the last layer which uses softmax. The error function was categorical cross-entropy, and the optimization algorithm was AMSGrad with learning rate 0.0002. Training was done with a batch size of 100 and dropout rate of 5%. The appropriate parameters for our dataset, including the sizes and number of the convolution blocks, as well as the choice of the learning algorithm and its parameters were determined empirically.

Note that another very often used approach for action recognition is to first transform the sequence of depth maps into features and then apply a classifier (see e.g., [17]). However, since our goal here was only to test the performance of basic methods on the new LMA dataset, for simplicity we decided to forgo the comparison with handcrafted features and instead do a comparison only with skeleton based methods (Section 4.2).

Transfer Learning

The action dataset that we used is relatively small. Transfer learning is an approach to do classifier learning that is frequently used for small datasets, and it has proved to be particularly successful for computer vision tasks. In transfer learning the classifier is first trained (*pretrained*) on a different but related dataset. The classifier parameters obtained after the pretraining step are then used as starting point to do additional training using the the target dataset.

For recognition using depth maps, we tested the same convolutional neural network structure as the one described above (Figure 4) for transfer learning. There are several approaches for how transfer learning can be done. One approach is to freeze the parameters of one part of the neural network, usually of the network layers closer to the input, and then use the target dataset to additionally train only the parameters of the last network layers. This is often called *feature-based transfer learning*. In our network we froze the parameters of the convolutional layers and additionally trained the last 3 fully connected layers.

A second variation of transfer learning that we also tested is not to freeze parameters but instead additionally train the whole network using the target dataset, which we will refer to as *fine tuning*. We used exactly the same network structure (DM3DCNN) for training from scratch, feature-based transfer learning and *fine tuning*.

#### 4.1.3. Pretraining Dataset: Simulating Depth Data

**Figure 5:**
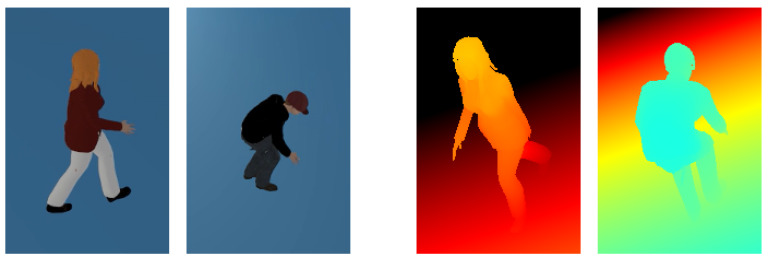
Simulation of human depth maps: (**left**) two examples of human models inside the simulator performing different actions; (**right**) the corresponding depth maps from a simulated Kinect sensor (showing just the part of the depth map close to the model).

An important factor in transfer learning is the dataset that is used for pretraining the classifier. For creating the pretraining dataset we used MORSE [55], a 3D simulator designed for robotic applications. The version of the simulator that we used was extended to allow the simulation of people [56]. We created 105 3D human models (Figure 5) featuring variations in size, age and gender, using the anatomically realistic human mesh generator MakeHuman [57]. Variations in clothing, as well as accessories such as hats or bag, were also added by manually editing the 3D models of clothes and adapting them to the body template of MakeHuman.

As a source of different human actions, we used the Carnegie Mellon University Motion Capture Database (CMU dataset) [58], which provides sequences of human data (skeleton data) captured using a motion capture system. The dataset contains more than 2500 samples of different actions, out of which we chose only the 1613 actions that were longer than 5 s. Using a MakeHuman plugin called MakeWalk we imported the motion captured action data into the skeletons of the generated 3D models. This was done while automatically adjusting for the differences of body proportions between the source and target. As a result we could simulate all the 105 human models doing all actions.

The MORSE simulator allows the simulation of 3D range sensor measurements. A single simulation consisted of loading one human model into an empty environment in the simulator and let it perform an action, while generating the corresponding depth image data from one simulated Kinect v2 sensor at 30 frames per second (Figure 5). Each simulation run lasted for the whole duration of the action. For each combination of model and action we made one simulation. At the beginning of each simulation the sensor position and orientation were randomly set, where the sensor’s pitch was restricted to looking downwards at a degree between 45${}^{\circ }$ and 135${}^{\circ }$, and the roll was limited to ±36${}^{\circ }$ from the horizontal alignment. Moreover, the distance of the sensor was chosen between 1 and 15 m with respect to a point in the center of the sensor’s image and at 1 m height from the floor. The starting position of the human model was within ±1 m in both *x* and *y* directions from that same center point, whereas the model’s orientation was randomly set. In the case the human moved out of the field of view of the sensor, we restarted the simulation for that model and action with new random conditions until a complete sample could be generated.

The total number of generated action samples was 169,365—one for each (model, action) combination. The depth maps were preprocessed in the same way as in the dataset with real people: the background (floor) was subtracted and the part belonging to the person was fit into a 64 × 64 image. As some actions were longer than the 5-s sequences used as input to DM3DCNN, in each epoch of the pretraining for each (model, action) combination one 5-second sequence was chosen at random.

Note that in a recent work Liu et al. [59] used a very similar technique to the one described here to synthetically generate both realistic RGB and depth data, which they used for improving human action recognition performance on publicly available datasets.

*Other datasets*: We also considered the use of other datasets for pretraining. In particular, the large NTU RGB+D dataset [6] seemed like a good option. However, we had difficulty preprocessing the depth maps in the same way as above. The dataset did not provide background information, and the point of view was changing across samples, which made it difficult to compute the background information. Although they did provide a range-of-interest view of the depth data, it included parts of other objects and we could not find an easy way to fix that. Therefore, we decided not to use it as pretraining dataset for depth map based recognition.

### 4.2. Skeleton Based Recognition

#### 4.2.1. Representation: Skeleton

In human action recognition one commonly used way to represent a person’s pose is to use a skeleton. It consists of the positions of the person’s body joints in 3D coordinates (or often 2D, if the skeleton is identified in a 2D image).

**Figure 6:**
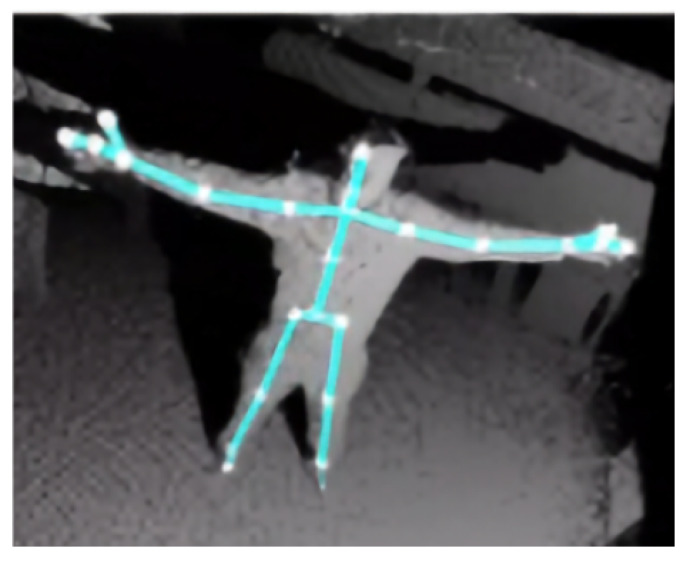
Example skeleton representation obtained from Microsoft Kinect v2 (overlaid on the depth image). The skeleton consists of 25 joints (shown as white circles), here connected with light blue lines for better visualization.

The Kinect sensors that we used during data collection provide also a skeleton data which contains 25 joints. One example is shown in Figure 6.

Similar to depth maps, with skeleton data we also used 5-second sequences as inputs to the classifiers. However, a skeleton data frame contains only 75 values (25 locations in 3D), compared to 4096 (64 × 64 pixels) values needed by the depth map. So instead of using every 5th frame, for skeleton data we used all 150 frames in the 5-second window. Thus, the input to the classifier was 150 frames of 75 values.

#### 4.2.2. Training Approaches

Training from Scratch

We tried training a number of classifiers from scratch. On the one hand, we used 2 classifiers which currently achieve top action recognition results on other skeleton datasets, with the expectation that they would do well on our dataset too. However, the number of parameters in those classifiers is large compared to the size of the dataset, so in addition we tried simpler classifiers as baseline methods, assuming that they could perform better.

*State-of-the-art skeleton data classifiers*: We tested two of the currently best performing neural network structures for skeleton data based action recognition: Spatial-Temporal Graph Convolutional Network (ST-GCN) [32] and Directed Graph Neural Networks (DGNN) [2].

In ST-GCN the authors first constructed a graph containing both spatial connections between neighboring joints as well as temporal connections. Then they used a graph convolutional neural network (GCN, see e.g., [60]) to perform classifications. At the time [32] was published this classifier had one of the best performances on a number of skeleton datasets.

Since ST-GCN was published several improvements have been proposed. The DGNN classifier is one of them, and at this moment, DGNN is in the top 5 reported performances in human action recognition on the NTU RGB+D dataset [6]. The original DGNN paper [2] employs a two-stream structure, where spatial and motion information are fed into two separate DGNN networks and then fused, which improves performance. However, since the reported performance of using both streams is only slightly better than when using only spatial information, in this work we only used the spatial stream DGNN, with the benefit of having a simpler structure.

For testing the ST-GCN and DGNN we used freely available implementations [61,62]. For training the networks we used the same parameters as in the original papers.

*Fully connected neural network*: We also tested a simple fully connected neural network. The structure of the network was optimized using grid search to find the one that achieves best performance on a subset of the full dataset. The final structure that we used consisted of 5 layers: 4 fully connected hidden layers which had 256 neurons and use rectified linear units (RELUs) as activation functions, and the output layer with 15 neurons and using the softmax activation function. Between the fully connected layers we used batch normalization and dropout (with dropout rate 50%). The loss function was sparse categorical cross-entropy, and the optimization algorithm used for training was Adagrad with learning rate 0.01.

*SVM*: Another commonly used type of classifier is the support vector machine (SVM). In our tests we have first applied the transformation of the input using an approximation of the Radial Basis Function (RBF) kernel [63] and then used an SVM with a linear kernel, since this approach scales better to large datasets than nonlinear SVMs.

Transfer Learning

Similarly to depth maps, we tested the transfer learning also for skeleton data, using the ST-GCN and DGNN classifiers. We utilized feature-based transfer learning, i.e. we pretrained the whole network using a different large dataset of skeleton actions (see below). After that we did additional learning only forof the last fully connected layer using the target dataset, while freezing the parameters of the rest of the network.

#### 4.2.3. Pretraining Datasets

For pretraining of the classifiers for transfer learning we used two different datasets.

**NTU dataset**: The NTU RGB+D dataset [6], which we already mentioned several times, is a dataset collected with human subjects using Kinect v2 sensors (NTU stands for Nanyang Technological University in Singapore). It contains 60 action classes which where performed by 40 different subjects and captured in 80 distinct camera viewpoints. The dataset contains 56,880 action sequences in total, saved in depth and skeleton, as well as RGB and infrared modalities.

To create the pretraining dataset we used the first 5 s from each action sequence. The reason is that some of the actions were short, and for these cases using the first 5 s insured that the whole action was contained. (We tried also randomly choosing the start of the sequence, but the performance did not improve.)

Note that the same research group now provides also an extended dataset with 120 actions [7]. However, as it was not available at the time we began our study, we used the one with 60 actions.

**CMU dataset**: The other dataset that we used is the CMU dataset (which we used to simulate depth maps for pretraining in Section 4.1.3). One important difference to the NTU dataset is that the skeleton model that the CMU dataset provides contains only 17 points. In order to use it as pretraining dataset we needed to convert it to the 25-point skeleton model that Kinect sensors provide. This was done by copying the matching skeleton points and approximately calculating the positions of the 8 missing joints.

The number of collected actions in the CMU dataset is more than 2000. However, they do not contain repetitions of the same actions performed by different subjects, like the NTU RGB+D dataset. To make up for that, we have regrouped the actions in the dataset based on the similarity of the textual descriptions of the actions. This was done partly automatically and partly through manual checks. We discarded all groups that had less than 5 actions, which resulted in about 60 groups of actions.

To further induce variation between the actions within each group, we have applied spatial transformations (about 100 different body sizes), as well as variations in the speed of the action (between 50% decrease and 50% increase in speed). Finally, for each sample we extracted five 5-s sequences by randomly selecting the starting point of the sequence. The thus obtained pretraining dataset from CMU data contained in total more than 130,000 sequences.

## 5. Evaluation

### 5.1. Evaluation Procedure

All the methods were evaluated on the Low-Moral Actions (LMA) dataset described in Section 3. The duration of all the saved sequences in the collected dataset was at least 13 s, so for the evaluation we used the first 13 s in each sequence. Kinect outputs 30 frames (depth maps and skeletons) per second, and for all the estimators the input consisted of 5 s of data. We used each possible 5-second interval within the 13-second sequence as one sample, which resulted is 240 samples per sequence. Both depth map and skeleton based methods used the same samples, where the first used raw depth data, whereas the latter used the skeletons extracted from depth data.

The accuracy was evaluated in a cross-subject experiment, using leave-one-subject-out cross-validation. That is, we trained a classifier on 19 subjects and validated on one, repeating the procedure for all 20 subjects and finally averaging the results. For transfer learning, the classifiers were first pretrained on the corresponding dataset, and the obtained weights were used as the starting point for training. The evaluation measure used is the recognition accuracy on the 15 action classes (percentage of correctly evaluated samples, which is also equivalent to the micro-F1 score).

### 5.2. Obtained Results

The results for all methods are summarized in Table 1. The methods which gave the highest recognition accuracy, for both depth maps and skeleton data, are highlighted in bold.

For both skeleton and depth maps, using transfer learning typically resulted in a better performance than learning from scratch. This is especially visible in the skeleton data results, where the accuracy when using transfer learning was considerably higher.

In the training from scratch with skeleton data, the two state-of-the-art neural network classifiers performed particularly poorly. The simpler baseline methods (SVM and fully connected NN ) performed somewhat better, but the accuracy was fairly low. However, when using transfer learning both the ST-GCN and DGNN classifiers performed well, and in fact DGNN pretrained on the NTU dataset achieved the best accuracy of all tested methods.

When using the DM3DCNN classifier on depth maps, the accuracy of directly training from scratch on the target dataset was much better. In fact, it was close to the result of transfer learning. The best classification result was obtained using fine tuning, and it was only slightly worse than the best classifier using skeleton data.

**Figure 7:**
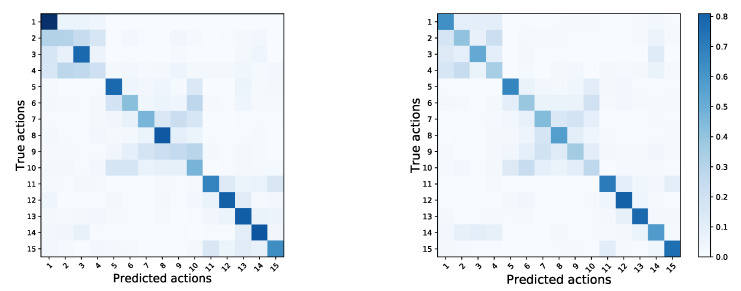
Confusion matrices for: (**left**)—DM3DCNN with fine tuning; (**right**)—DGNN with pretraining on the NTU RGB+D dataset.

Figure 7 shows the confusion matrices for the classifiers that obtained best accuracy, both for depth maps and skeleton data. Comparing the matrices we can see that both methods show similar error patterns. In particular, it seems that misclassification mostly happened within the three action groups, e.g., actions while standing (1 to 4) were often confused with other actions while standing, but not with actions while walking (5 to 10) or interactive actions (11 to 15). This is expected, as the actions within the action groups were indeed more similar to each other.

We also report the training speeds for the two best classifiers. DM3DCNN took about 32.5 h for pretraining, whereas the fine tuning around 4.5 h. The DGNN pretraining took 12.75 h, and the transfer learning part about 1 h. All training times were obtained on a desktop PC with an Intel Core i9-7940X (14 core) CPU and Nvidia GeForce GTX 1080 Ti graphic card.

## 6. Discussion

The obtained results seem to confirm a number of our intuitions for training classifiers on relatively small datasets:

- blindly applying a classifier which had top performance on a large dataset directly to a smaller dataset might not work well
- transfer learning can be a good way to train such classifiers, and in general it will outperform training from scratch
- for pretraining, datasets containing realistic variations, such as the ones collected from a large number of subjects, are better

Directly using depth maps for action recognition has received much less attention than the use of skeleton representation. However, as our results show, a depth maps based classifier can achieve comparable performance.

Even just training from scratch already gave a relatively good result on the classifier using depth maps, but interestingly, the same was not true for the skeleton data based classifiers. We initially guessed that the problem might be that they had too many parameters to be trained on the LMA dataset, as it is much smaller than the NTU dataset. (ST-GCN has about 3 million, and DGNN about 4 million parameters; in comparison, DM3DCNN has only about 370,000). But we also tried training downscaled versions of these classifiers, with the number of parameters comparable to DM3DCNN, yet the results did not improve. We hypothesize that the difference might be due to the compression of information when skeletons are extracted—e.g., from two different sensors we obtain a very similar skeleton, just rotated in space. On the other hand, the depth maps from different views are more diverse. But this would need to be confirmed with additional experiments.

The results for skeleton based classifiers show that pretraining on the NTU dataset gave better results than using the CMU dataset. Even though the CMU dataset was about 2.3 times larger, the skeleton it provided was only an approximation of the skeleton from a Kinect sensor. Moreover, the variations in the datasets (in speed and person sizes) were artificially induced, unlike the variations between real human subjects in the NTU dataset. This confirms the value of the efforts of creating large datasets of real human actions.

The improvement when using pretraining with simulated data on the DM3DCNN was not very large. It would thus be interesting to know how much simulated data is actually enough for the pretraining. A small preliminary test has shown that using 20 simulated models from the dataset instead of all 105 gives 53.73% accuracy, and using only 10 models gives 53.35% accuracy. This shows that even using a much smaller dataset for pretraining, with less variation between simulated models, may be good enough. However, a more thorough study is left for future work.

## 7. Conclusions

Motivated by the aim to recognize low-moral behavior in public spaces, we studied the recognition of relatively rare actions for which data cannot be easily collected. We collected a new dataset of actions, which contains a high percentage of low-moral actions. We ran several action recognition methods, to provide a baseline recognition result for the dataset, as well as compare: (a) depth maps vs. skeleton representation; (b) training from scratch vs. transfer learning; (c) different datasets for pretraining. The results confirmed that, for this problem, transfer learning can indeed improve the performance of the classifiers. Both depth maps and skeleton data were shown to be able to achieve similar performance. Hopefully this will spur more research into depth map based methods for action recognition, which are currently much less studied then skeleton based ones.

## Figures and Tables

**Table 1:** Recognition accuracy (Top-1) on the Low-Moral Actions (LMA) dataset. (FCNN = fully connected neural network; for transfer learning on skeleton data the dataset used for pretraining is denoted in squared brackets.) For the two highlighted best methods we also denote the Top-5 accuracy in brackets.

	Training from Scratch:		Transfer Learning:	
**Deptd map**	DM3DCNN	47.27%	DM3DCNN (feature-based t. l.)	44.47%
			**DM3DCNN (fine tuning)**	**54.37%** (90.04%)
**Skeleton**	5-layer FCNN	18.92%	ST-GCN [NTU]	53.52%
	SVM	21.40%	ST-GCN [CMU]	47.84%
	ST-GCN	5.00%	**DGNN [NTU]**	**56.58%** (91.39%)
	DGNN	11.60%	DGNN [CMU]	51.78%

## Supplementary Materials

Both skeleton and depth maps of the LMA Dataset will be made available online at http://www.robot.soc.i.kyoto-u.ac.jp/en/research/low-moral-action-dataset/.
